# Supplementary figures and images for: Association between virtual visits and health outcomes of people living with HIV: A cross-sectional study
Source: PLoS One. 2025 May 28;20(5):e0315880. doi: 10.1371/journal.pone.0315880 (PMC12118912; doi:10.1371/journal.pone.0315880)

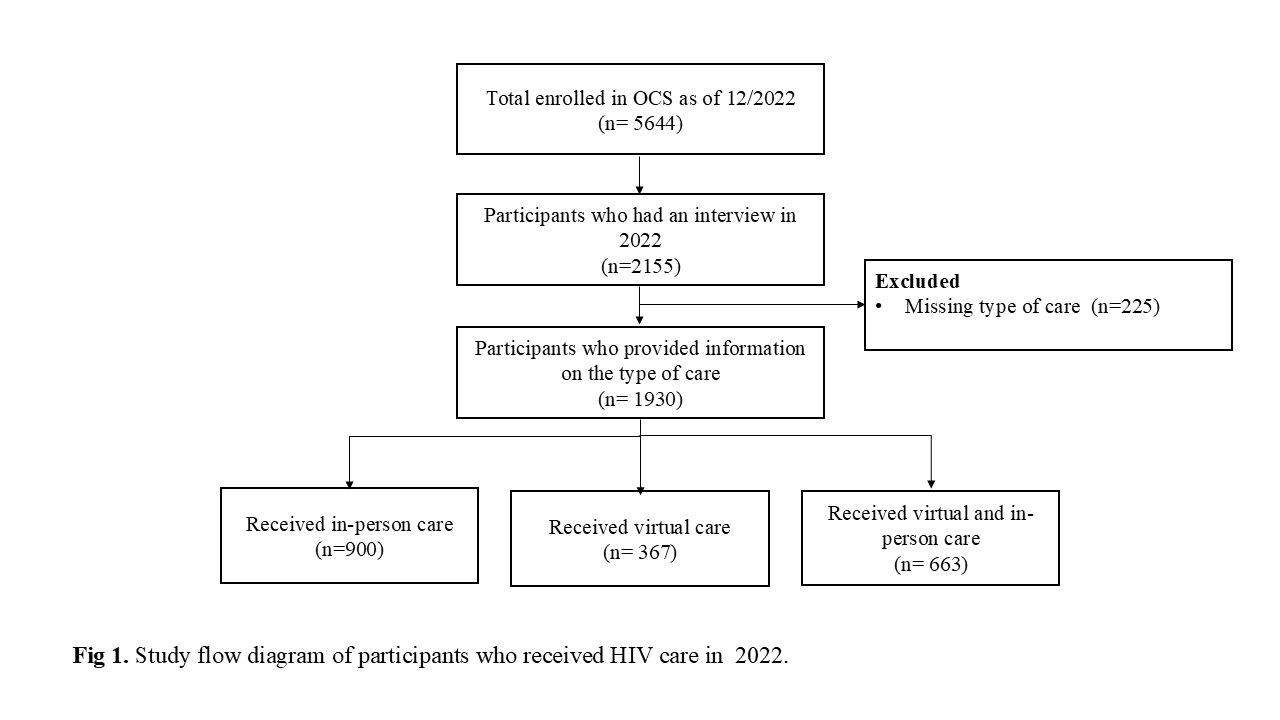

Supplement: S1 Fig — (TIF) [file pone.0315880.s001.tif]
