# Supplementary material for: Association between virtual visits and health outcomes of people living with HIV: A cross-sectional study
Source: PLoS One. 2025 May 28;20(5):e0315880. doi: 10.1371/journal.pone.0315880 (PMC12118912; doi:10.1371/journal.pone.0315880)
Supplement: S1 Table — (DOCX) [file pone.0315880.s002.docx]

| S 1 Table: Multivariable subgroup regression analysis of HIV-related outcomes of people receiving care in Ontario, Canada | | | | |
| --- | --- | --- | --- | --- |
| Variable | Unadjusted Effect Estimates (95%CI) | p-value | Adjusted Effect Estimates  (95% CI) | p-value |
| *Adherence to ART (OR)* | | | | |
| Subgroup analysis by lockdown-ref: no lockdown (n=1161) | | | | |
| *Ease on lockdown (n=36)* | | | | |
| In-person care | (1.00) ref |  | 1.00 (ref) |  |
| Virtual and in-person | 1.98 (0.46, 8.54) | 0.361 | 1.81 (0.40, 6.09) | 0.442 |
| Virtual | 0.83 (0.06, 11.81) | 0.892 | 0.86 (0.06, 2.36) | 0.914 |
| *Lockdown (n=682)* | | | | |
| In-person care | (1.00) ref |  | 1.00 (ref) |  |
| Virtual and in-person | 1.66 (1.04, 2.64) * | 0.030 | 1.61 (0.99, 5.01) | 0.055 |
| Virtual | 1.53 (0.89, 2.65) | 0.133 | 1.49 (0.85*,* 4.45) | 0.167 |
| Subgroup analysis by physicians’ preference based on viral load (ref: suppressed/stable VL ≤ 40 copies/mL) | | | | |
| In-person care | 1.00 (ref) |  | 1.00 (ref) |  |
| Virtual and in-person | 0.61 (0.32, 1.17) | 0.142 | 0.55 (0.28, 1.09) | 0.094 |
| Virtual | 0.29 (0.10, 0.83) * | 0.022 | 0.28 (0.09, 0.86) * | 0.032 |
| Subgroup analysis based on sex and orientation (reference category: male MSM) | | | | |
| *Subgroup analysis by female* | | | | |
| In-person care | 1.00 (ref) |  | 1.00 (ref) |  |
| Virtual and in-person | 1.12 (0.66, 1.92) | 0.668 | 1.14 (0.66, 2.00) | 0.634 |
| Virtual | 1.03 (0.52, 2.04) | 0.923 | 1.01 (0.50, 2.03) | 0.991 |
| *Subgroup analysis by male non-MSM* | | | | |
| In-person care |  |  |  |  |
| Virtual and in-person | 1.03 (0.58, 1.81) | 0.9243 | 0.95 (0.53, 1.71) | 0.873 |
| Virtual | 0.73 (0.36, 1.46) | 0.369 | 0.67 (0.32, 1.39) | 0.282 |
| Subgroup analysis based on race/ethnicity (reference category: White) | | | | |
| *Subgroup analysis by ACB* | | | | |
| In-person care | 1.00 (ref) |  | 1.00 (ref) |  |
| Virtual and in-person | 0.84 (0.50, 0.53) | 0.530 | 0.90 (0.52, 2.00) | 0.693 |
| Virtual | 0.75 (0.36, 0.45) | 0.420 | 0.76 (0.37, 1.60) | 0.473 |
| *Subgroup analysis by other races* | | | | |
| In-person |  |  |  |  |
| Virtual and in-person | 0.99 (0.54, 1.82) | 0.969 | 1.09 (0.58, 2.06) | 0.784 |
| Virtual | 1.41 (0.71, 2.81) | 0.326 | 1.323 (0.65, 2.71) | 0.440 |
| Subgroup analysis based on stigma status (ref: no stigma) | | | | |
| In-person care | 1.00 (ref) |  | 1.00 (ref) |  |
| Virtual and in-person | 1.20 (0.65, 2.20) | 0.554 | 1.03 (0.47, 2.26) | 0.940 |
| virtual | 1.67 (0.51, 5.53) | 0.397 | 2.92 (0.66, 12.89) | 0.156 |
| *Viral load (OR)* | | | | |
| Subgroup analysis by lockdown (ref-no lockdown) | | | | |
| Ease on lockdown (n=36) | NA |  | NA |  |
| In-person care | 1.00 (ref) |  | 1.00 (ref) |  |
| Virtual and in-person | NA |  | NA |  |
| Virtual | NA |  | NA |  |
| *Lockdown (n=682)* | | | | |
| In-person care | 1.00 (ref) |  | 1.00 (ref) |  |
| Virtual and in-person | 1.35 (0.55, 3.12) | 0.532 | 1.61 (0.67, 3.84) | 0.288 |
| Virtual | 0.92 (0.30, 2.85) | 0.898 | 1.00 (0.31, 3.12) | 0.995 |
| Subgroup analysis by physicians’ preference based on viral load (ref: suppressed/stable VL ≤ 40) | | | | |
| In-person care | 1.00 (ref) |  | 1.00 (ref) |  |
| Virtual and in-person | NA |  | NA |  |
| Virtual | NA |  | NA |  |
| Subgroup analysis based on sex and orientation (reference category: male MSM) | | | | |
| *Subgroup analysis by female* | | | | |
| In-person care | 1.00 (ref) |  | 1.00 (ref) |  |
| Virtual and in-person | 0.82 (0.36, 1.85) | 0.646 | 1.04 (0.44, 2.43) | 0.920 |
| Virtual | 0.64 (0.18, 2.22) | 0.495 | 0.64 (0.17, 2.32) | 0.499 |
| *Subgroup analysis by male non-MSM* | | | | |
| In-person care | 1.00 (ref) |  | 1.00 (ref) |  |
| Virtual and in-person | 1.19 (0.51, 2.70) | 0.689 | 1.09 (0.46, 2.63) | 0.831 |
| Virtual | 0.44 (0.16, 1.21) | 0.125 | \| 0.45 (0.15, 1.35) \| \| --- \| | 0.157 |
| Subgroup analysis based on race/ethnicity (reference category: White) | | | | |
| *Subgroup analysis by ACB* | | | | |
| In-person care | 1.00 (ref) |  | 1.00 (ref) |  |
| Virtual and in-person | 2.77 (0.62, 2.04) | 0.478 | 1.38 (0.67, 3.03) | 0.410 |
| Virtual | 2.38 (0.29, 0.71) | 0.730 | 0.72 (0.58, 2.12) | 0.533 |
| *Subgroup analysis by other races* | | | | |
| In-person | 1.00 (ref) |  | 1.00 (ref) |  |
| Virtual and in-person | 2.43 (0.97, 5.88) * | 0.057 | 2.08 (0.79, 5.26) | 0.135 |
| Virtual | 0.411 (0.17, 1.03) | 0.225 | 2.12 (0.58, 7.70) | 0.253 |
| *Subgroup analysis by stigma (ref: no stigma)* | | | | |
| In-person | 1.00 (ref) |  | 1.00 (ref) |  |
| Stigma (bot | 1.03 (0.47, 2.27) | 0.934 | 0.82 (0.26, 2.56) | 0.732 |
| virtual | 1.27 (1.04, 6.25) | 0.774 | 1.04 (0.10, 1.04) | 0.969 |
| *MCS* | | | | |
| Subgroup analysis by lockdown (ref: no lockdown) | | | | |
| *Ease on lockdown (n=36)* | | | | |
| In-person care | 0 (ref) |  | 0 (ref) |  |
| Virtual and in-person | -3.26 (-11.48, 4.96) | 0.442 | -1.93 (-7.74, 3.88) | 0.512 |
| Virtual | 8.15 (-6.68, 22.98) | 0.281 | 2.56 (-7.90, 13.03) | 0.634 |
| *Lockdown (n=682)* | | | | |
| In-person care | 0 (ref) |  | 0 (ref) |  |
| Virtual and in-person | 0.53 (-2.05, 3.10) | 0.693 | -0.01(-1.89, 1.87) | 0.990 |
| Virtual | -0.09 (-3.15, 2.96) | 0.954 | 1.79 (-0.39, 3.95) | 0.111 |
| Subgroup analysis by physicians’ preference based on viral load (ref: suppressed*/stable VL ≤ 40)* | | | | |
| In-person care | 0 (ref) |  | 0 (ref) |  |
| Virtual and in-person | -5.60 (-9.46, -1.75) * | 0.000 | -3.75 (-6.51, -0.99) * | 0.015 |
| Virtual | -0.94 (-6.23, 4.23) | 0.731 | 0.35 (-4.164, 3.46) | 0.867 |
| Subgroup analysis based on sex and orientation (reference category: male MSM) | | | | |
| *Subgroup analysis by female* | | | | |
| In-person care | 0 (ref) |  | 0 (ref) |  |
| Virtual and in-person | -1.69 (-4.69, 1.32) | 0.274 | -0.07 (-2.27, 2.13) | 0.953 |
| Virtual | -2.56 (-6.40, 12.28) | 0.192 | -0.94 (-3.70, 1.81) | 0.504 |
| *Subgroup analysis by male non-MSM* | | | | |
| In-person care | 0 (ref) |  | 0 (ref) |  |
| Virtual and in-person | -1.56 (-4.75, 1.63) | 0.344 | 0.35 (-1.94, 2.65) | 0.767 |
| Virtual | -4.39 (-8.34, 0.44) * | 0.031 | -0.17 (3.07, 2.74) | 0.913 |
| Subgroup analysis based on race/ethnicity (reference category: White) | | | | |
| *Subgroup analysis by ACB* | | | | |
| In-person care | 0 (ref) |  | 0 (ref) |  |
| Virtual and in-person | -1.76 (-4.70, 1.18) | 0.242 | -0.68 (-2.80, 1.44) | 0.534 |
| Virtual | 3.34 (-0.58, 7.25) | 0.091 | 2.10 (-0.71, 4.92) | 0.145 |
| *Subgroup analysis by other races* | | | | |
| In-person care | 0 (ref) |  | 0 (ref) |  |
| Virtual and in-person | 2.93 (-0.50, 6.37) | 0.090 | 1.03 (-1.45, 3.50) | 0.426 |
| Virtual | 0.81 (-3.15, 4.77) | 0.691 | -0.08 (-2.93, 2.77) | 0.963 |
| *Stigma* | | | | |
| Virtual and in-person | 0.74 (-2.55, 4.03) | 0.657 | 1.02 (-1.77, 3.83) | 0.471 |
| virtual | 6.65 (0.16, 13.14) | 0.579 | 2.738 (-2.58, 8.05) | 0.311 |
| *PCS* | | | | |
| Subgroup analysis by lockdown (ref: no lockdown) | | | | |
| *Ease on lockdown (n=36)* | | | | |
| In-person care | 0 (ref) |  | 0 (ref) |  |
| Virtual and in-person | -2.72 (-10.49, 4.76) | 0.477 | -1.81 (-8.63, 5.00) | 0.605 |
| Virtual | 0.40 (-13.02, 13.81) | 0.956 | -1.02 (-13.17, 11.13) | 0.875 |
| *Subgroup analysis by lockdown (n=682)* | | | | |
| In-person care | 0 (ref) |  | 0 (ref) |  |
| Virtual and in-person | -0.16 (-2.61, 2.26) | 0.905 | 0.04 (-2.21, 2.30) | 0.974 |
| Virtual | -1.62 (-4.46, 1.21) | 0.263 | -1.21 (-3.84, 1.41) | 0.363 |
| Subgroup analysis by physicians’ preference based on viral load (ref: suppressed/stable VL ≤ 40) | | | | |
| In-person care | 0 (ref) |  | 0 (ref) |  |
| Virtual and in-person | 0.57 (-2.89, 4.03) | 0.755 | 1.25 (-1.91, 4.40) | 0.445 |
| Virtual | -2.61 (-7.84, 2.61) | 0.334 | -1.23 (-6.01, 3.54) | 0.613 |
| Subgroup analysis based on sex and orientation (reference category: male MSM) | | | | |
| *Subgroup analysis by female* | | | | |
| In-person care | 0 (ref) |  | 0 (ref) |  |
| Virtual and in-person | -2.30 (-5.03, 0.43) | 0.101 | -1.82 (-4.34, 0.70) | 0.166 |
| Virtual | -2.48 (-6.02, 1.06) | 0.174 | -1.39 (0.41, 1.92) | 0.413 |
| *Subgroup analysis by male non-MSM* | | | | |
| In-person care | 0 (ref) |  | 0 (ref) |  |
| Virtual and in-person | -2.75 (-5.70, 0.20) | 0.075 | -1.46 (-4.19, 1.28) | 0.304 |
| Virtual | -2.59 (-6.34, 1.16) | 0.186 | -1.09 (-4.59, 2.41) | 0.546 |
| Subgroup analysis based on race/ethnicity (reference category: White) | | | | |
| *Subgroup analysis by ACB* | | | | |
| In-person care | 0 (ref) |  | 0 (ref) |  |
| Virtual and in-person | -1.20 (-3.95, 1.54) | 0.395 | -0.76 (-3.25, 1.73) | 0.554 |
| Virtual | 1.06 (-2.57, 4.68) | 0.573 | 0.37 (-2.99, 3.72) | 0.834 |
| *Subgroup analysis by other races* | | | | |
| In-person care | 0 (ref) |  | 0 (ref) |  |
| Virtual and in-person | 1.66 (-1.50, 4.82) | 0.302 | 0.27 (-2.65, 3.18) | 0.866 |
| Virtual | 0.45 (-3.22, 4.12) | 0.813 | -0.25 (-3.62, 3.12) | 0.885 |
| Subgroup analysis by stigma (ref: no stigma) | | | | |
| *In-person care* | 0 (ref) |  | 0 (ref) |  |
| Virtual and in-person | 0.62 (-2.63, 3.87) | 0.707 | 0.80 (-2.58, 4.19) | 0.641 |
| Virtual | 1.19 (-5.49, 7.86) | 0.726 | 0.03 (-6.64, 6.72) | 0.991 |
| *Significant  ^a^ Adjusted | | | | |
