# Supplementary material for: Association between virtual visits and health outcomes of people living with HIV: A cross-sectional study
Source: PLoS One. 2025 May 28;20(5):e0315880. doi: 10.1371/journal.pone.0315880 (PMC12118912; doi:10.1371/journal.pone.0315880)
Supplement: Attachment — Submitted filename: Response to Reviewer.docx [file pone.0315880.s005.docx]

The Editor,

PLOS ONE Journal

Dear Editor,

Thank you for the opportunity to revise our manuscript (PONE-D-24-55188R1), titled *“Association between virtual visits and health outcomes of people living with HIV: A cross-sectional study,”* for consideration in **PLOS ONE**.

We sincerely appreciate the reviewers’ time and thoughtful feedback. We have carefully addressed each comment and incorporated the suggested changes to enhance the clarity, quality, and scientific rigor of our manuscript.

Below, we provide a detailed response to each point raised. The revised manuscript and a marked-up version highlighting the changes are attached. We believe these revisions align with the journal’s standards and welcome any further feedback.

Thank you again for your consideration.

Sincerely,

Nadia Rehman

| **Reviewer’s Comments** | **Authors’ Response** | **Page No.** |
| --- | --- | --- |
| **Introduction** | | |
| Abbreviation PLHIV shows for the first time, needs the full term here. | We have made this correction by introducing the full term followed by the abbreviation. The revised text now reads:  “Evidence on the effect of virtual care on health outcomes for people living with HIV (PLHIV) is limited. While some studies focus on retention improvement rather than health outcomes, findings are mixed.” | Page 5, line 78 |
| The consistency of abbreviation: page 8, in the part of secondary outcomes, “QOL” and “QoL” are both used. | Thank you for pinpointing this mistake. We have corrected it by introducing the abbreviation for quality of life as QoL. We have fixed the error through out the manuscript. The revised text now reads:  “QoL was assessed using the Short Form 12-item Health Survey (version 2), which includes the Mental Component Summary Score (MCS) and the Physical Component Summary Score (PCS). Both scores are reported separately, with higher values indicating better QoL in their respective domains.” | Page 8, line 139  Page 3,  line 35,  Page 6, line 87 & 91 |
| The consistency of a number of digits in results and tables: page 11, some percentages keep 1 digit on the decimal, and some are rounded numbers. Tables one and two have the same issue. | We appreciate the reviewer’s careful attention to detail. We have made the necessary corrections in both tables, and all percentages are now presented with one decimal place. | Page 11 |
| The variable “virtual care” is significant in adjusted model, needs a “*” after the CI. | We have made the correction and added an asterisk to virtual care in Table 2. | Page 15, table 2 |
